# Comparison of the Immunogenicity of HIV-1 CRF07_BC Gag Antigen With or Without a Seven Amino Acid Deletion in p6 Region

**DOI:** 10.3389/fimmu.2022.850719

**Published:** 2022-04-05

**Authors:** Minchao Li, Yue Yuan, Pingchao Li, Zhaomin Deng, Ziyu Wen, Haiying Wang, Fengling Feng, Huachun Zou, Ling Chen, Shixing Tang, Caijun Sun

**Affiliations:** ^1^ School of Public Health (Shenzhen), Shenzhen Campus of Sun Yat-sen University, Shenzhen, China; ^2^ State Key Laboratory of Respiratory Disease, Guangzhou Institutes of Biomedicine and Health (GIBH), Chinese Academy of Sciences, Guangzhou, China; ^3^ Guangdong Provincial Key Laboratory of Tropical Disease Research, School of Public Health, Southern Medical University, Guangzhou, China; ^4^ Key Laboratory of Tropical Disease Control (Sun Yat-Sen University), Ministry of Education, Guangzhou, China

**Keywords:** HIV-1, CRF07_BC, p6Δ7, Gag, immunogenicity

## Abstract

HIV-1 CRF07_BC-p6Δ7, a strain with a seven amino acid deletion in the p6 region of the Gag protein, is becoming the dominant strain of HIV transmission among men who have sex with men (MSM) in China. Previous studies demonstrated that HIV-1 patients infected by CRF07_BC-p6Δ7 strain had lower viral load and slower disease progression than those patients infected with CRF07_BC wild-type strain. However, the underlying mechanism for this observation is not fully clarified yet. In this study, we constructed the recombinant DNA plasmid and adenovirus type 2 (Ad2) vector-based constructs to express the HIV-1 CRF07_BC Gag antigen with or without p6Δ7 mutation and then investigated their immunogenicity in mice. Our results showed that HIV-1 CRF07_BC Gag antigen with p6Δ7 mutation induced a comparable level of Gag-specific antibodies but stronger CD4^+^ and CD8^+^ T-cell immune responses than that of CRF07_BC Gag (07_BC-wt). Furthermore, we identified a series of T-cell epitopes, which induced strong T-cell immune response and cross-immunity with CRF01_AE Gag. These findings implied that the p6^Gag^ protein with a seven amino acid deletion might enhance the Gag immunogenicity in particular cellular immunity, which provides valuable information to clarify the pathogenic mechanism of HIV-1 CRF07_BC-p6Δ7 and to develop precise vaccine strategies against HIV-1 infection.

## Introduction

The epidemic of HIV-1 infection remains a global health crisis. According to the report issued by the Joint United Nations Program on HIV/AIDS (UNAIDS), 37.7 million people were living with HIV-1 including 10.2 million untreated, 1.5 million new HIV-1 infections, and 680,000 AIDS-related deaths at the end of 2020. Although antiretroviral therapy (ART) can effectively control viral replication in AIDS patients, the withdrawal of ART will inevitably lead to a rapid rebound of plasma viremia because of the stable and long-lived latent virus reservoirs (1, 2). Thus, HIV-1 patients have to receive life-long ART therapy. An effective HIV-1 vaccine is therefore considered the most cost-effective intervention to eventually terminate the HIV-1 spread. However, the extraordinary genetic diversity, latent viral reservoirs, and lack of the immune correlates of protection against HIV-1 infection are the major challenges for developing an effective HIV-1 vaccine. So far, hundreds of HIV-1 vaccine trials have been studied, including the AIDSVAX trial (protein subunit vaccine, aimed to produce the neutralizing antibodies), STEP trial (viral vectored-HIV vaccine, aimed to elicit T-cell immune responses), and RV144 trial (combined immunization with ALVAC-HIV/AIDSVAX, aimed to induce a balanced immunity with both humoral and cellular responses) (3, 4). Among them, the RV144 trial remains the only one to demonstrate a positive signal with an estimated efficacy of 31.2% (5). Therefore, it is of great significance to develop the next generation of HIV-1 prophylactic vaccine to confer better immune protection or therapeutic vaccines to achieve a functional cure.

Circulating recombinant form 07_BC strain (CRF07_BC) is a genotype of HIV-1 with a clade C backbone in which several Thailand variant fragments of clade B into structural and accessory genes were inserted (6). CRF07_BC originated in Yunnan and spread rapidly in Western China and then nationwide (7). Subsequently, HIV-1 CRF07_BC had caused an epidemic among Taiwan injection drug users in 2004 (8, 9). Full-length genomic analysis of CRF07_BC revealed that there were some deletions (7–11 amino acids) in the p6 (Gag) and p6 (Pol) proteins (10). Our previous study showed that the prevalence of 07_BC-p6Δ7 strain constantly increased and exceeded 90% in the men who have sex with men (MSM) population infected by HIV-1 CRF07_BC in China (11), indicating that 07_BC-p6Δ7 is becoming the dominant strain among MSM in China. Notably, the deletion sequences YPX(n)L in the Gag p6 region mediated the interaction with ALG-2 interacting protein X (Alix) protein, which functions not only in multivesicular body (MVB) biogenesis but also in apoptosis, endocytosis, and cytokinesis pathways (12). In addition, p6Δ7 mutation reduced the protease-mediated processing of Gag/Gag-Pol polyproteins and decreased the incorporation of Vpr protein in the virus particle, leading to a reduced viral load and replication, slower viral maturation processes, and an increased proportion of immature virus particles (13, 14). These characteristics of CRF07_BC-p6Δ7 might be the consequence of co-adaption between the host and the virus. The lower viral load and slower disease progression seem to be beneficial to the host survival, but they also prolong the virus survival time, and the unapparent symptom before clinical diagnosis might also provide more opportunity to spread this strain among the population and thus poses a serious public health threat.

It is of note that the Gag protein is a well-known antigen to stimulate cellular responses to control HIV-1 replication and has thus been extensively studied as an ideal target for developing the HIV-1 vaccine. HIV-1-specific T cell responses play pivotal roles in controlling viral infection (15, 16). The strong cellular response not only protected acute HIV-1 infection by reducing initial peak viremia to viral set point (17, 18) but also existed in long-term non-progressors with chronic HIV-1 infection (19, 20). Moreover, HIV-1-specific T-cell immune responses induced by different HIV-1 proteins, especially the specific cytotoxic T lymphocyte (CTL) response against the Gag protein, could control HIV-1 replication to maintain a low steady-state viremia in several independent cohort studies (21–24). Considering that the Gag-specific T-cell immune responses can contribute to controlling the viral load and disease progression, we therefore speculate whether the p6Δ7 mutation in the Gag protein might change the Gag-specific immune responses and then affect the disease progression. We therefore address this critical issue in this study using 15-mer overlapping peptides spanning the entire sequence of the Gag protein to identify the specific T-cell epitopes through ELISpot as well as intracellular cytokine staining (ICS) assay.

## Materials and Methods

### Vaccine and Peptide Preparation

The HIV-1 Gag protein sequences were obtained from the National Center for Biotechnology Information (NCBI) including HIV-1 CRF07_BC-p6Δ7 (GenBank: AHA50525.1) and CRF01_AE (GenBank: AFV34153.1). The Gag sequence was optimized according to the preferred codon usage of mammalian cells as previously described (25) and chemically synthesized by Invitrogen (Carlsbad, CA, USA). Subsequently, the CRF07_BC gag full length with the supplemental deletion of seven amino acids in the p6 region (07_BC-wt) was obtained using overlap extension PCR with two pairs of specific primers designed based on the CRF07_BC-p6Δ7 sequence (Primer: A1:5′CTTAAGCTTACCATGGGCGCC, B1:5′CCCTCTAGATTTAGGGCAGCAGGGG, A2:5′GTACAGCTCCTTGTCAATGGGCTCCTGCTTCTGGGATGGGG, B2:5′CCCATTGACAAGGAGCTGTACCCCATCACCTCCCTGAAGTC). The codon-optimized Gag gene was then cloned into the pVAX-1 expression vector (Invitrogen) and adenovirus type 2 (Ad2) vector. Recombinant adenoviruses were generated using homologous recombination methods as our previous study described (26).

In this study, a total of 121 of 15-mer peptides overlapping by 11 amino acid residues and spanning the gene sequence of 07_BC-wt were designed using the software PeptGen (http://www.hiv.lanl.gov/content/sequence/PEPTGEN/peptgen.html) and synthesized (purity: >85%, GeneScript, China). The peptide pool was divided into 12 pools, each of which contained 10 peptides, and the last peptide pool contained 11 peptides (**Supplementary Table 1**). All of the peptides were dissolved in dimethyl sulfoxide (DMSO) at 0.4 mg/ml.

### Western Blotting Analysis

To confirm the expression of the target protein, human embryonic kidney (HEK) 293 cells in 6-well plates were transfected with pVAX-01AEgag, pVAX-07BCgag-p6Δ7, pVAX-07BCgag-wt, or pVAX-empty at 2 μg per well or infected with rAd2-01AEgag, rAd2-07BCgag-p6Δ7, rAd2-07BCgag-wt, or rAd2-empty at 0.2 TCID50 per well for 48 h at 37°C. Subsequently, cells were lysed, and the proteins were separated by sodium dodecyl sulfate–polyacrylamide gel electrophoresis (SDS-PAGE) under denaturing and reducing conditions. Proteins were transferred to the polyvinylidene difluoride (PVDF) membrane (Bio-Rad, Hercules, CA, USA). After being blocked for 1 h with 5% skim milk in Tris buffered saline with Tween-20 (TBST) the membrane was incubated overnight with a rabbit monoclonal anti-Gag antibody (Abcam, Cambridge, UK) and a mouse monoclonal anti-GAPDH antibody (Abcam) at 4°C. The membrane was washed and then incubated with horseradish peroxidase-conjugated anti-rabbit IgG antibody or anti-mouse IgG antibody at a 1:5,000 dilution (Abcam) for 2 h. Finally, the membranes were developed with a chemiluminescent horseradish peroxidase (HRP) substrate (Invitrogen). The expression of GAPDH was also examined in parallel as an internal control.

### Animal Experiments

Female BALB/c mice aged 6–8 weeks were randomly allocated into five groups (n = 10 per group). Group 1 received 100 μl of PBS per mouse served as the negative control. Each mouse of groups 2 to 5 was intramuscularly injected with 50 μg of the DNA plasmid dissolved in 100 μl of PBS in the right quadriceps of the leg at weeks 0 and 2 and then boosted intramuscularly with 1 × 10^9^ vp of the corresponding adenoviral vector vaccine in the right quadriceps of the leg at weeks 4. At weeks 3 and 6, five mice in each group were anesthetized with inhaled isoflurane and sacrificed by cervical dislocation. Then, the splenocytes were obtained and subjected to subsequent immunological analyses.

### IFN-γ ELISpot Assay

IFN-γ ELISpot assay was performed to detect cellular immune response using freshly isolated mouse splenic lymphocytes as previously described (25). Briefly, 96-well plates (Merck Millipore, Burlington, MA, USA) were washed and coated with purified rat anti-mouse IFN-γ monoclonal antibody U-CyTech, Netherlands at 4°C overnight. Mouse splenic lymphocytes were isolated using mouse lymphocyte separation solution (Dakewe Biotech Co., Ltd., Shenzhen, China) and seeded in the plates at 3 × 10^5^ per well. Then each group was incubated with the peptide pool at 4 μg/ml, while DMSO served as a negative control. After incubation for 24 h, the plates were incubated with biotinylated detection antibodies (U-CyTech) and developed with alkaline phosphatase-conjugated streptavidin (BD Biosciences, San Jose, CA, USA) and NBT/BCIP reagent (Thermo Fisher Scientific, Waltham, MA, USA). Finally, the spots were counted with an ELISpot reader (Bioreader 4000, BIO-SYS, Karben, Germany).

### Intracellular Cytokine Staining

The antigen-specific cytokine secreted by lymphocytes was also detected by ICS assay as previously described (27). Briefly, mouse splenic lymphocytes were seeded in the 96-well plates at 2 × 10^6^ per well and incubated with the peptide pool at 4 μg/ml for 2 h at 37°C. Then brefeldin A (BD Biosciences) was added and incubated for 16 h at 37°C. The cells were harvested and stained with CD3-PerCP-Cy-5.5, CD4-APC, and CD8-PE (BD Biosciences) for 30 min protected from light; then added with cytofix/cytoperm (BD Biosciences) to permeabilize for 30 min protected from light; and stained with IFN-γ-FITC, IL-2-APC-Cy7, and TNF-α-PE-Cy7 (BD Biosciences) for 1 h protected from light at 4°C. Samples were analyzed using the FACSAria instrument with FlowJo software (version 7.6).

### Identification of Potential T-Cell Epitope

To identify the potential CTL epitopes, the single 15-mer peptide in the peptide pools of the 07BC Gag protein was analyzed using the NetMHCpan-4.0 EL 4.0 algorithm with a size range of 8- to 11-mers amino acid (28, 29). The most frequent major histocompatibility complex (MHC) class I alleles in BALB/c mice, including H-2-Dd, H-2-Kd, and H-2-Ld, were selected as a model to analyze the potential CTL epitopes. The epitope was determined based on the Percentile Rank, which was lower than 0.5 (most likely to be considered as high-affinity epitopes). In addition, 01AE was aligned with 07BC-p6Δ7 using NCBI blast software (**Supplementary Figure 1**) and predicted CTL epitope through NetMHCpan-4.0 EL 4.0 algorithm.

### Statistical Analysis

Flow cytometric data were analyzed using FlowJo version 7.6. Statistical analyses and graphical presentations were conducted with GraphPad Prism version 8.0. One-way ANOVA was used for the comparison among multiple (>2) groups, and a two-tailed unpaired Student’s t-test was used for comparison between two groups (07BC-p6Δ7 and 07BC-wt). Data were expressed as mean ± SEM, and p-values of less than 0.05 were deemed statistically significant. *p < 0.05; **p < 0.01; ***p < 0.001; NS, no significance.

## Results

### Construction of Recombinant DNA and Ad2 Vectors Carrying Various Gag Genes

**Figure 1:**
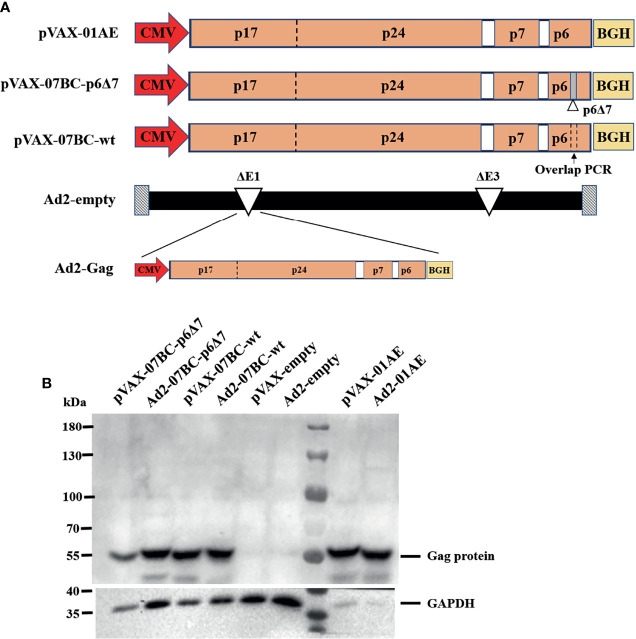
Construction and characteristic of the DNA vectors and Ad2-based vectors carrying the Gag protein of different HIV-1 subtypes. **(A)** Diagrammatic sketch of constructing pVAX-1 and Ad2 vectors carrying various Gag genes under CMV promoter and the gene structure of Gag. There are seven amino acid deletions in Gag protein encoded by 07BC-p6Δ7, while the deletions were supplemented in 07BC-wt through overlap PCR. **(B)** Western blotting analysis of the expression of various Gag proteins in HEK293 cells in 6-well plates transfected with plasmids encoding a codon-modified Gag sequence (pVAX-01AE, pVAX-07BC-p6Δ7, or pVAX-07BC-wt, 2 μg per well) or infected with recombinant Ad2 virus (Ad2-01AE, Ad2-07BC-p6Δ7, or Ad2-07BC-wt, 0.2 TCID50 per well). pVAX-empty plasmid and Ad2-empty virus were used as the negative control. The GAPDH blot examined in parallel served as an internal control.

As described in the *Materials and Methods*, we successfully obtained the genes of CRF07_BC-p6Δ7, CRF07_BC gag full length (07_BC-wt), and CRF01_AEgag. To assess their immunogenicity, we constructed the recombinant pVAX-1 vector and adenovirus type 2 vector carrying various Gag genes under the control of the CMV promoter (**Figure 1A**). The Ad2 vector with deletion of E1 and E3 genes was replication-defective, and Gag gene was inserted into the E1 region. Then recombinant Ad2 was rescued and propagated in HEK293 cells, which can provide E1 protein to support the replication of this E1-deleted virus. Western blotting results showed that both the DNA constructs and Ad2 constructs appropriately expressed the corresponding Gag protein with expected molecular weight (55 kDa) (**Figure 1B**).

### Immunization With Recombinant DNA and Ad2 Vectored Constructs Effectively Elicited Antigen-Specific Immune Responses Targeting Different Regions of Gag Protein

**Figure 2:**
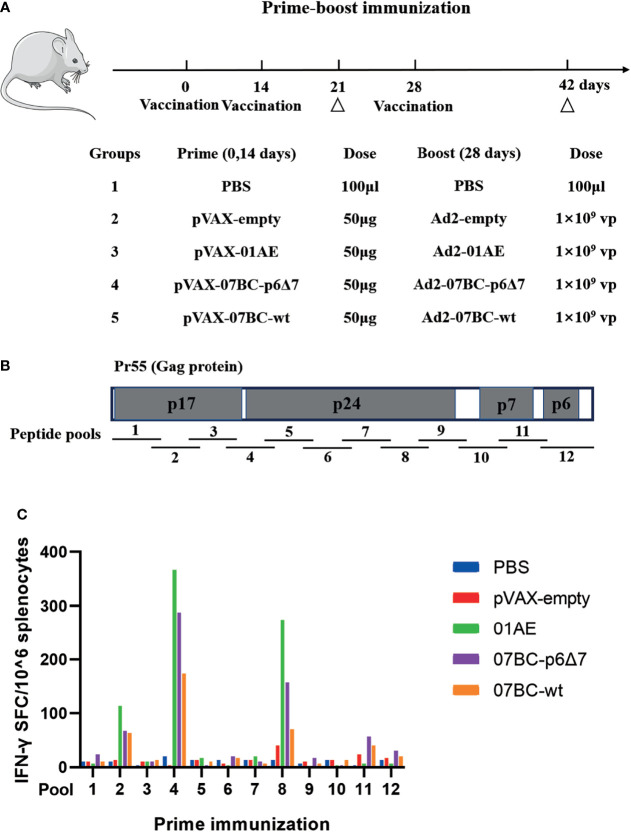
Strong antigen-specific IFN-γ response against different regions of Gag protein after primary immunization in mice. **(A)** Vaccination strategy to evaluate the immunogenicity of various Gag proteins. Female BALB/c mice aged 6–8 weeks were randomly allocated into 5 groups (n = 10 per group). Each mouse was intramuscularly injected with 50 μg of the DNA plasmid dissolved in 100 μl of phosphate-buffered saline (PBS) at weeks 0 and 2 and then boosted intramuscularly with 1 × 10^9^ vp of the corresponding adenoviral vector at weeks 4. Group PBS served as the negative control. “Δ” represents the time-point of sacrifice (5 mice per group) and sample collection. **(B)** The structure of Gag protein and the distribution of the peptide pools. Gag gene encodes HIV-1 viral core proteins including p17, p24, and p15. p15 is further cleaved into nucleocapsid proteins p7 and p6. The peptide pool was divided into 12 pools, each of which contained 10 peptides, and the last peptide pool contained 11 peptides. **(C)** Cellular immune responses of different peptide pools after primary immunization with DNA vaccine. The IFN-γ ELISpot assay was used to detect the cellular immune response following stimulation with peptide pools after DNA-based vaccine immunization on day 21. Median responses (n = 5) in BALB/c mice following vaccination are shown by the number of spot-forming cells (SFCs) in one million splenic lymphocytes.

To investigate the immunogenicity of the Gag protein with or without p6Δ7 mutation, 6- to 8-week-old female BALB/c mice were immunized as shown in **Figure 2A**, and then the antigen-specific immune responses were evaluated. Previous studies demonstrated that the Gag-specific T-cell immune response, but not Gag-specific antibody, was associated with lower viremia and better HIV-1 control (21, 22). Since there was a comparable level of Gag-specific antibodies in both the 07BCgag-p6Δ7 group and 07BCgag-wt group, we focused on the Gag-specific T-cell immune response in the following study. In our study, the peptide pool covering the full length of the Gag protein was divided into 12 sub-pools (pool 1–pool 12) to stimulate mouse spleen lymphocytes (**Figure 2B**). After immunization with recombinant DNA and Ad2 vectored constructs, the peptide pools covering different regions of the Gag protein elicited T-cell immune responses with different intensities (**Figure 2C**). There were obvious T-cell immune responses induced by pool 2, pool 4, pool 8, pool 11, and pool 12, but not for the remaining sub-peptide pools. Among them, the immune responses elicited by the stimulation of peptide pool 2, pool 4, and pool 8, which are located at the p17 and p24 regions of the Gag protein, were stronger than those of pool 11 and pool 12, which are located at the p7 and p6 regions of the Gag protein. Of note, there were stronger T-cell immune responses in the 07_BC-p6Δ7 group when compared with the 07_BC-wt group, suggesting that the seven amino acid deletion in the p6 region might mainly affect the immunogenicity of T-cell epitopes in the Gag protein.

### Identification of Cross-Reactive Cytotoxic T Lymphocyte Epitopes Between 07_BC and 01_AE

**Figure 3:**
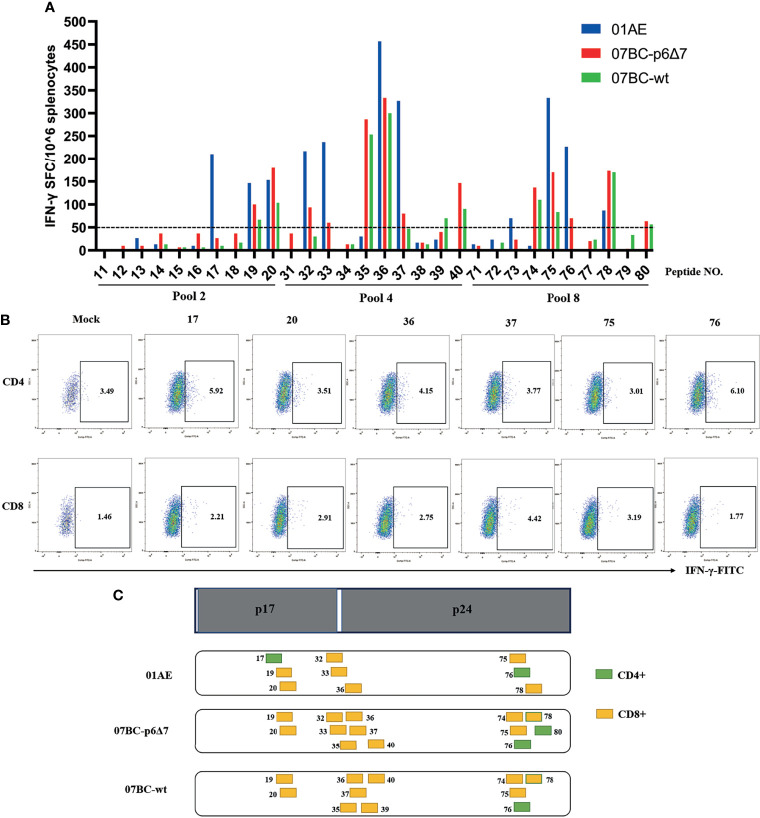
Specific T-cell responses induced by Ad2-based vaccine and identification of the T-cell epitope. After Ad2 boost vaccination, mice were sacrificed, and the splenocytes from mice were restimulated *ex vivo* with peptide pools. The background-subtracted median responses (n = 5) in BALB/c mice following vaccination are shown. **(A)** Mouse splenic lymphocytes isolated after boost vaccination were stimulated with individual overlapping 15-mer peptides (pool 2, pool 4, and pool 8) in IFN-γ ELISpot assay. The dotted line represents the cutoff value. **(B)** Peptides that successfully induced responses above cutoff values in IFN-γ ELISpot assays were then tested for their ability to induce IFN-γ production in T cells by intracellular cytokine staining (ICS) assays. **(C)** Epitope mapping analysis of the CD8^+^ and CD4^+^ T-cell epitopes is represented by orange and green boxes, respectively.

We then mapped the T-cell epitopes in the mice immunized with different vaccine constructs. IFN-γ ELISpot assay was performed to identify the vaccine-induced T-cell response towards each peptide in pool 2, pool 4, and pool 8 by setting the cutoff value of 50 spot-forming cells (SFCs)/10^6^ cells as a positive response (30). After the background was subtracted, the median value is shown in **Figure 3A**, and a series of T-cell epitopes were identified (**Table 1**). After the identification of overall T-cell responses, the positive responses against specific peptide pools were then characterized by CD4^+^ or CD8^+^ T-cell subsets for ICS assay (**Figure 3B** and **Supplementary Figure 2**). The frequency of IFN-γ-positive CD4^+^ and CD8^+^ T cells is shown in **Table 1**. In our study, twelve peptides could stimulate a positive CD8^+^ T-cell response, and three peptides could stimulate a positive CD4^+^ T-cell response. Except for peptide 73, the most positive peptides confirmed by IFN-γ ELISpot assay could also be recognized by the ICS assay of CD4^+^ or CD8^+^ T-cell subsets.

**Table 1:** Identification of the T-cell epitope.

Peptide sequence	Peptide no.^a^	SFC/10^6b^	CD3^+^CD4^+^IFN-γ (%)^c^	CD3^+^CD8^+^IFN-γ (%)^d^	Subtype
QPALQTGTEELRSLF	P2-17	210	2.43	0.75	01AE
EELRSLFNTVATLYC	P2-19	147	0.13	1.01	01AE
		100	0.14	0.96	07BC-p6Δ7
		67	N/D	0.33	07BC-wt
SLFNTVATLYCVHTG	P2-20	153	0.02	1.45	01AE
		180	0.39	2.05	07BC-p6Δ7
		103	N/D	0.32	07BC-wt
VSQNYPIVQNIQGQM	P4-32	217	0.58	2.10	01AE
		93	N/D	1.96	07BC-p6Δ7
YPIVQNIQGQMVHQP	P4-33	237	0.34	0.77	01AE
		60	1.05	2.16	07BC-p6Δ7
GQMVHQPISPRTLNA	P4-35	287	N/D	2.07	07BC-p6Δ7
		253	N/D	0.63	07BC-wt
HQPISPRTLNAWVKV	P4-36	457	0.66	1.29	01AE
		333	N/D	1.40	07BC-p6Δ7
		300	N/D	0.98	07BC-wt
SPRTLNAWVKVVEEK	P4-37	327	0.28	2.96	07BC-p6Δ7
		80	N/D	0.58	07BC-wt
VKVVEEKAFSPEVIP	P4-39	70	N/D	1.20	07BC-wt
EEKAFSPEVIPMFSA	P4-40	147	0.45	1.60	07BC-p6Δ7
		90	N/D	0.43	07BC-wt
PFRDYVDRFFKTLRA	P8-73	70	N/D	N/D	01AE
YVDRFFKTLRAEQAT	P8-74	137	0.66	1.83	07BC-p6Δ7
		110	N/D	1.12	07BC-wt
FFKTLRAEQATQDVK	P8-75	333	N/D	1.73	01AE
		170	0.43	1.66	07BC-p6Δ7
		83	N/D	1.02	07BC-wt
LRAEQATQDVKNWMT	P8-76	227	2.61	0.31	01AE
		70	2.82	N/D	07BC-p6Δ7
DVKNWMTDTLLVQNA	P8-78	87	0.10	0.97	01AE
		173	0.16	0.78	07BC-p6Δ7
		170	N/D	0.71	07BC-wt
TLLVQNANPDCKTIL	P8-80	63	1.16	0.68	07BC-p6Δ7
		57	N/D	N/D	07BC-wt

Median responses (n = 5) in BALB/c mice following vaccination are shown.

N/D, not detected; SFC, spot-forming cell.

a The peptide number of the overlapping peptide pool.

b Background-subtracted IFN-γ SFC/10^6^ splenocytes.

c Background-subtracted frequency of responding CD3^+^CD4^+^IFN-γ cells as percentage of all CD3^+^CD4^+^ splenocytes.

d Background-subtracted frequency of responding CD3^+^CD8^+^IFN-γ cells as percentage of all CD3^+^CD8^+^ splenocytes.

We further constructed the epitope mapping to analyze the breadth of the cellular response after vaccination (**Figure 3C**). Of note, although the overlapping peptide pool was constructed based on the sequence of the 07BC Gag protein, it also stimulated the splenocytes from 01AE-immunized mice to produce high levels of IFN-γ secretion. Therefore, we aligned 01AE (GenBank: AFV34153.1) and 07BC-p6Δ7 (GenBank: AHA50525.1) sequences using NCBI blast software (**Supplementary Figure 1**) and found that the similarity of amino acid sequences between 01AE and 07BC-p6Δ7 was 82%. To identify the cross-reactive T-cell epitopes, the single 15-mer peptide in the peptide pools of the 07BC Gag protein was analyzed using the NetMHCpan-4.0 EL 4.0 algorithm with a size range of 8- to 11-mer amino acid. As shown in **Table 2**, we predicted 6 cross-reactive CTL epitopes, which were most likely to be high-affinity epitopes. Furthermore, these cross-reactive CTL epitopes were conserved and maintained the recognition of CD8^+^ T cells restricted by different MHC-I molecules even with a single amino acid substitution. Consistent with our prediction, peptide 36 (HQPISPRTLNAWVKV) contains two epitopes and thus effectively induced more than 300 SFCs/10^6^ in IFN-γ ELISpot assay that targets different MHC-I molecules, suggesting that this peptide might induce extensive cross-reactive Gag-specific T-cell responses for effective HIV-1 control.

**Table 2:** Prediction of the T-cell epitopes with cross-reactive T-cell responses between 07BC and 01AE.

Peptide sequence	Peptide no.	Predicted epitope (07BC)^a^	Predicted epitope (01AE)^b^	Predicted MHC subtype^c^	T-cell response^d^
EELRSLFNTVATLYC	P2-19	SLFNTVATL	SLFNTVATL	H-2-Kd	CD8^+^
SLFNTVATLYCVHTG	P2-20	SLFNTVATL	SLFNTVATL	H-2-Kd	CD8^+^
VSQNYPIVQNIQGQM	P4-32	YPIVQNIQGQM	YPIVQNAQGQM	H-2-Ld	CD8^+^
YPIVQNIQGQMVHQP	P4-33	YPIVQNIQGQM	YPIVQNAQGQM	H-2-Ld	CD8^+^
HQPISPRTLNAWVKV	P4-36	ISPRTLNAW	VSPRTLNAW	H-2-Dd	CD8^+^
		HQPISPRTL	HQPVSPRTL	H-2-Kd/H-2-Dd	CD8^+^
SPRTLNAWVKVVEEK	P4-37	SPRTLNAWV	SPRTLNAWV	H-2-Ld	CD8^+^
DVKNWMTDTLLVQNA	P8-78	NWMTDTLLV	NWMTETLLV	H-2-Kd	CD8^+^

The epitope was predicted using the NetMHCpan-4.0 EL 4.0 algorithm and a size range of 8- to 11-mers amino acid. The epitope was determined based on the Percentile Rank, which was lower than 0.5.

MHC, major histocompatibility complex; CTL, cytotoxic T lymphocyte.

a Predicted epitope in Gag protein of 07BC.

b Predicted epitope in Gag protein of 01AE

c The mice MHC subtype with high binding affinity to CTL epitope.

d The type of T-cell response.

### The Seven Amino Acid Deletion in p6 Region Contributed to the Increased Cellular Immune Response

**Figure 4:**
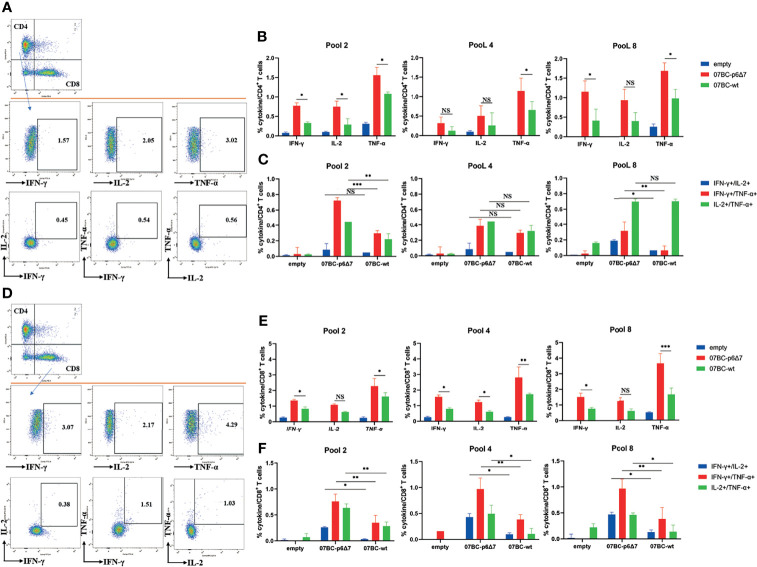
Assessment of Gag-specific cellular immunity elicited by Ad2 vaccine through intracellular cytokine staining (ICS) assay. The ability of polyfunctional CD4^+^ and CD8^+^ T-cell populations from immunized mice to secrete IFN-γ, TNF-α, and IL-2 cytokines in response to Gag peptide pools stimulation was assessed. The background-subtracted median responses (n = 5) in BALB/c mice following vaccination are shown. Gating strategy for flow cytometric scatter plots to analyze the frequency of the single or multiple cytokine(s)-positive CD4^+^ **(A)** or CD8^+^ **(D)** T cells. Frequencies of CD4^+^ **(B)** or CD8^+^ **(E)** T cells secrete IFN-γ, TNF-α, and IL-2 cytokines specific to various Gag antigens after adenoviral-based immunization. Further analysis was performed to show subpopulations of double cytokines-secreting CD4^+^ T cells **(C)** or CD8^+^ T cells **(F)**. Data were expressed as mean ± SEM, and p-values of less than 0.05 were deemed statistically significant.*p < 0.05; **p < 0.01; ***p < 0.001; NS, no significance.

To further study the poly-functionality of T-cell populations after immunization with various Gag modalities, we performed the multi-parameter ICS assay to assess the Gag-specific cellular immunity. The frequency of CD4^+^ T-cell subsets producing one cytokine (IFN-γ, TNF-α, and IL-2) or more cytokines (IFN-γ^+^/IL-2^+^, IFN-γ^+^/TNF-α^+^, and IL-2^+^/TNF-α^+^) was analyzed using the exclusive gating strategy (**Figure 4A**). Statistical analysis demonstrated that the frequency of CD4^+^ T cells secreting one cytokine or dual cytokines in the 07BC-p6Δ7-immunized mice was significantly higher than that of the 07_BC-wt group, especially in response to the stimulation of pool 2 and pool 8 peptides (**Figures 4B, C**). In addition, a similar observation was also found in the CD8^+^ T cells secreting one cytokine or dual cytokines (**Figures 4D–F**).

## Discussion

In this study, we evaluated the immunogenicity of the HIV-1 CRF07_BC Gag antigen with or without a seven amino acid deletion in the p6 region in mice. Our results demonstrated that CRF07_BC-p6Δ7 induced a stronger T-cell immune response than CRF07_BC-wt. The difference between the two sequences is the seven amino acids in the Gag p6 region, which are critical for binding with Alix protein. Mutations at the Alix-binding site of the p6 region led to impaired HIV-1 replication and the decreased efficiency of viral budding in various cell types (12). A previous study revealed that the expression of Galectin-3 was upregulated in response to HIV-1 CRF07_BC infection (31), and the endogenous Galectin-3 facilitated the binding of Alix-Gag p6 complex to promote HIV-1 budding (32). However, this promoting effect could be disrupted because of the seven amino acid deletion in the Gag p6 region (33). Thus, p6Δ7 might play an important role in controlling the lower viral load and slower disease progression. In addition, Galectin-3 negatively regulates the adhesion between T cell and antigen-presenting cells (APCs) by destabilizing the immunological synapse, and the possible mechanism is through modulating Alix’s function to downregulate the T-cell function (34). This is consistent with our results that 07BC Gag with a seven amino acid deletion induced higher magnitude IFN-γ in ELISpot assay and higher frequency of Gag-specific (p17 and p24) cytokine including IFN-γ, IL-2, and TNF-α, indicating that the seven amino acid deletion in the p6 region might promote the immunogenicity of Gag antigen by disrupting the interactions between Galectin-3 and Alix-Gag p6 complex. Since it is well known that the enhancement of Gag-specific T-cell immune response can contribute to controlling the viral load and disease progression, our findings provide a new explanation for the abovementioned observation.

The cross-recognition of viral epitopes by CD8^+^ T cells is related to viral control during HIV-1 infection and is thus vital to develop a vaccine strategy that can elicit cross-reactive T-cell responses to target the conserved regions of the viral antigens, which is an important requirement for an effective vaccine against HIV-1 variants (35, 36). In our study, we also identified seven 15-mer peptides, which can induce a high magnitude of IFN-γ secretion in ELISpot assay, and further predicted six CD8^+^ T-cell epitopes located at the conserved p17 and p24 region of the 01AE Gag and the 07BC Gag, indicating that there might be a strong cross-reactive immune response between the 01AE Gag antigen and the 07BC Gag antigen. Among them, peptide 36 (HQPISPRTLNAWVKV) induced a robust T-cell immunity, and further analysis demonstrated that it contained two CTL epitopes with the potential to target different MHC-I molecules. HIV-1 CRF07_ BC was a recombinant virus of the B′ and C subtypes, which probably shared 50% CTL epitopes in Gag and reverse transcriptase (RT) with prototype B strain (6). Interestingly, the cross-reactive T-cell responses in HIV CRF01_AE and B′ had been observed (37). This evidence supported that there were cross-reactive T-cell responses between 01AE and 07BC, which is consistent with our findings.

To our best knowledge, this is the first study to investigate the immunogenicity of HIV-1 CRF07_BC with or without seven amino acid deletions in the p6 region of the Gag protein, and we found that the seven amino acid deletion in the p6 region contributed to the increased CD4^+^ and CD8^+^ T immune response against Gag antigen. These data should be helpful to clarify the mechanism why HIV-1 CRF07_BC-p6Δ7 patients have a lower viral load and slower disease progression than other patients. However, further study is needed to distinguish the clinical course and immunological response in patients infected with CRF07_BC-p6Δ7 compared to CRF07_BC strains. In addition, our findings will also provide insights for the development of Gag-based vaccine strategies against HIV-1 infection.

## Data Availability Statement

The raw data supporting the conclusions of this article will be made available by the authors, without undue reservation.

## Ethics Statement

All animal experiments were conducted in the Animal Experimental Center of the Guangzhou Institutes of Biomedicine and Health, Chinese Academy of Sciences. This experimental protocol was approved by the Institutional Animal Care and Use Committee of Guangzhou Institute of Biomedicine and Health (Aprroved number:2020053; Date of acceptance: April 3, 2020; Duration: Four months).

## Author Contributions

CS conceived and designed the experiments. ML, YY, PL, ZD, ZW, and HW performed the experiments. ML, ZD, ZW, and CS analyzed the data. ML and CS wrote the manuscript. FF, HZ, LC, and ST contributed to the resource and discussion. All of the authors read the final version of the manuscript.

## Funding

This work was supported by the National Natural Science Foundation of China (81971927), the Natural Science Foundation of Guangdong Province (2019A1515110458), China Postdoctoral Science Foundation (2020T130150ZX), the National Science and Technology Major Project of China (2018ZX10731101-002), and the Science and Technology Planning Project of Shenzhen City (20190804095916056, JSGG20200225152008136, JCYJ20200109142601702).

## Conflict of Interest

The authors declare that the research was conducted in the absence of any commercial or financial relationships that could be construed as a potential conflict of interest.

## Publisher’s Note

All claims expressed in this article are solely those of the authors and do not necessarily represent those of their affiliated organizations, or those of the publisher, the editors and the reviewers. Any product that may be evaluated in this article, or claim that may be made by its manufacturer, is not guaranteed or endorsed by the publisher.
