# Supplementary material for: Comparison of the Immunogenicity of HIV-1 CRF07_BC Gag Antigen With or Without a Seven Amino Acid Deletion in p6 Region
Source: Front Immunol. 2022 Apr 5;13:850719. doi: 10.3389/fimmu.2022.850719 (PMC9017423; doi:10.3389/fimmu.2022.850719)
Supplement: Supplementary file 1 [file DataSheet_1.pdf]

Supplementary Figure

Alignment of sequence: 01AE (GenBank: AFV34153.1) with 07BC-p6Δ7 (GenBank: AHA50525.1)

Similarity: 409/497(82%)

```
Query  1    MGARASVLSGGKLDAWEKIRLRPGGKKKYRMKHLVWASRELERFALNPGLLETAEGCQQI  60
            MGARASVL GGKLD WEKIRLRPGGKK+YR+KHLVWASRELERFALNPGLLET+EGC+QI
Sbjct  1    MGARASVLRGGKLDKWEKIRLRPGGKKRYRLKHLVWASRELERFALNPGLLETSEGCKQI  60

Query  61   IEQLQSTLKTGSEELKSLFNTVATLWCVHQRIDVKDTKEALDKIEEVQNKSQQKTQQAAA  120
            + QLQ  L+TG+EEL+SLFNTVATL+CVH  IDV+DTKEALDKIEE QNK QQKTQQA
Sbjct  61   MNQLQPALQTGTEELRSLFNTVATLYCVHTGIDVRDTKEALDKIEEEQNKIQQKTQQAKE  120

Query  121  GTGSSSKVSQNYPIVQNAQGQMVHQPVSPRTLNAWVKVVEEKGFNPEVIPMFSALSEGAT  180
               +  KVSQNYPIVQN QGQMVHQP+SPRTLNAWVKVVEEK F+PEVIPMFSALSEGAT
Sbjct  121  ---ADRKVSQNYPIVQNIQGQMVHQPISPRTLNAWVKVVEEKAFSPEVIPMFSALSEGAT  177

Query  181  PQDLNMMLNIVGGHQAAMQMLKETINEEAADWDRTHPVQAGPIPPGQIREPRGSDIAGTT  240
            PQDLN MLN VGGHQAAMQ+LK+TINEEAA+WDR HPV AGPI PGQ+REPRGSDIAGTT
Sbjct  178  PQDLNTMLNTVGGHQAAMQILKDTINEEAAEWDRLHPVHAGPIAPGQMREPRGSDIAGTT  237

Query  241  STLQEQIAWMTNNPPIPVGDIYKRWIILGLNKIVRMYSPVSILDIRQGPKEPFRDYVDRF  300
            STLQEQI WMT+NPP+PVGDIYKRWIILGLNKIVRMYSP SILDI+QGPKEPFRDYVDRF
Sbjct  238  STLQEQITWMTSNPPVPVGDIYKRWIILGLNKIVRMYSPTSILDIKQGPKEPFRDYVDRF  297

Query  301  YKTLRAEQATQEVKNWMTETLLVQNANPDCKSILKALGTGATLEEMMTACQGVGGPSHKA  360
            +KTLRAEQATQ+VKNWMT+TLLVQNANPDCK+IL+ALG GA++EEMMTACQGVGGPSHKA
Sbjct  298  FKTLRAEQATQDVKNWMTDTLLVQNANPDCKTILRALGPGASIEEMMTACQGVGGPSHKA  357

Query  361  RVLAEAMSQAQQTNIMMQKGIFKGPKRI-KCFNCGKEGHLARNCRAPRKKGCWKCGKEGH  419
            RVLAEAMSQ   + IMMQ+  F+G KRI KCFNCGKEGH+A+NCRAPRKKGCWKCGKEGH
Sbjct  358  RVLAEAMSQTN-SAIMMQRSNFRGSKRIVKCFNCGKEGHIAKNCRAPRKKGCWKCGKEGH  416

Query  420  QMKGCTERQANFLGKIWPSNKGRPGNFPQSRPEPTAPPAEDWGMGEEIASLPKQEQKGKD  479
            QMK CTERQANFLGKIWPS+KGRPGNF QSRPEPTAPP E +  GEEI + P Q+Q+
Sbjct  417  QMKDCTERQANFLGKIWPSHKGRPGNFLQSRPEPTAPPEESFRFGEEITT-PSQKQE---  472

Query  480  QRPPSVSLKSLFGNDPL  496
               P  SLKSLFGNDPL
Sbjct  473  ---PITSLKSLFGNDPL  486
```

Figure S1. Amino acid alignment of 01AE and 07BC-p6Δ7. 01AE (Query) and 07BC-p6Δ7 (Sbjct) were aligned using NCBI blast software.

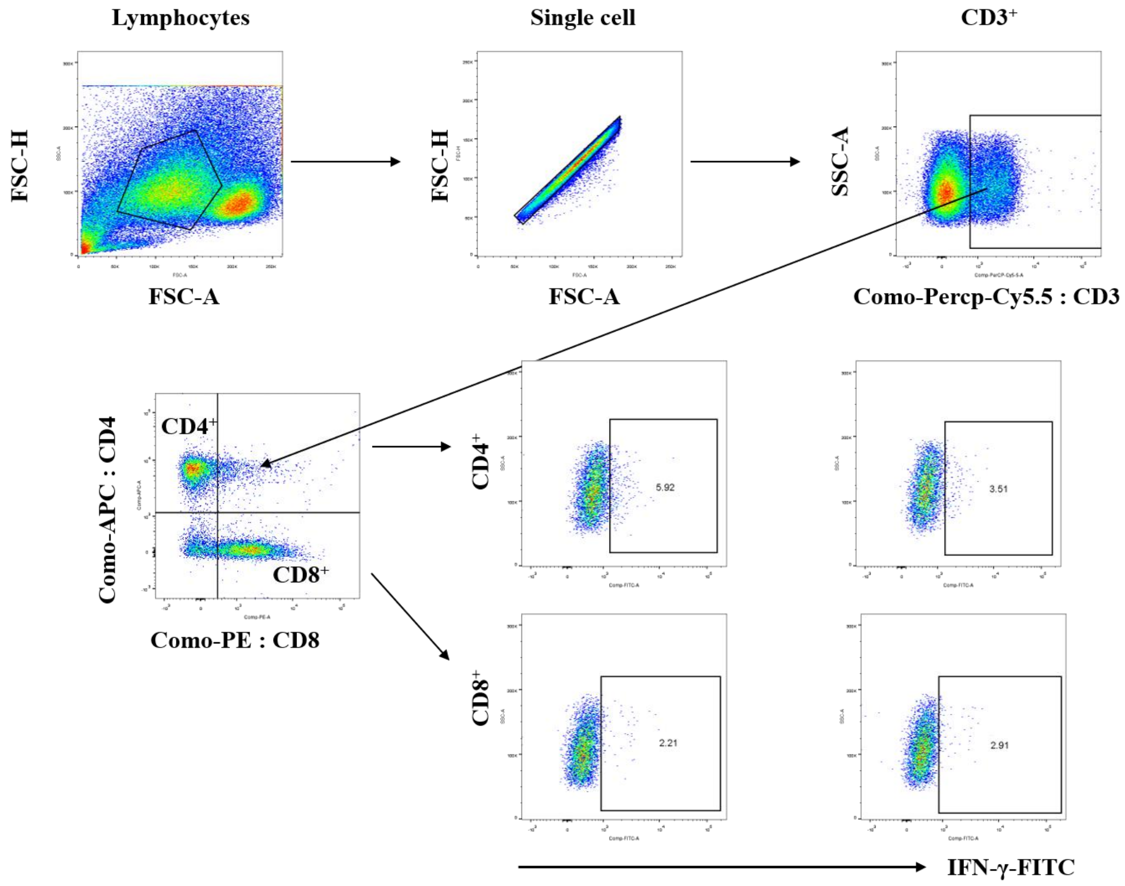

Figure S2. Examples of phenotype determination of T cells recognizing optimal peptides in Table 1 and Figure 3B.
